# Myocardial tissue tagging with cardiovascular magnetic resonance

**DOI:** 10.1186/1532-429X-11-55

**Published:** 2009-12-21

**Authors:** Monda L Shehata, Susan Cheng, Nael F Osman, David A Bluemke, João AC Lima

**Affiliations:** 1 Department of Radiology, Johns Hopkins University School of Medicine, Baltimore, MD, USA; 2 Division of Cardiology, Department of Medicine, Johns Hopkins University School of Medicine, Baltimore, MD, USA; 3 Department of Radiology, National Institutes of Health, Bethesda, MD, USA

## Abstract

Cardiovascular magnetic resonance (CMR) is currently the gold standard for assessing both global and regional myocardial function. New tools for quantifying regional function have been recently developed to characterize early myocardial dysfunction in order to improve the identification and management of individuals at risk for heart failure. Of particular interest is CMR myocardial tagging, a non-invasive technique for assessing regional function that provides a detailed and comprehensive examination of intra-myocardial motion and deformation. Given the current advances in gradient technology, image reconstruction techniques, and data analysis algorithms, CMR myocardial tagging has become the reference modality for evaluating multidimensional strain evolution in the human heart. This review presents an in depth discussion on the current clinical applications of CMR myocardial tagging and the increasingly important role of this technique for assessing subclinical myocardial dysfunction in the setting of a wide variety of myocardial disease processes.

## Introduction

Heart failure (HF) is the result of advanced myocardial dysfunction and continues to be a leading cause of morbidity and mortality in developed nations. In the United States alone, an estimated 5.3 million adults carry the diagnosis of HF and the disease prevalence continues to escalate with aging of the population. In addition to conferring a significant burden of illness to affected individuals, management of HF also imposes enormous expense to the health care system [1]. Therefore, the ability to identify individuals at risk for developing HF - those who might benefit from targeted preventive interventions - would be of immense value.

To this end, some of the most promising approaches to improved risk stratification involve using imaging modalities to detect early myocardial dysfunction. Assessment of global ventricular function - and its reduced indices, such as ejection fraction, are clearly strong predictors of future HF and poor prognosis. However, global measures are insensitive to reductions in regional performance, where even a normal ejection fraction can obscure significant underlying regional dysfunction. Thus, measures of regional function, such as quantification of myocardial strain and torsion, have emerged as more accurate tools for defining degrees of myocardial disease. Abnormalities in these measures of regional function can also serve as a more specific marker of subclinical myocardial dysfunction. Several techniques have been used to quantify regional myocardial function. Nuclear techniques [2] can provide information on global and regional wall motion but with limited spatial and temporal resolution. Tissue Doppler imaging [3]and Speckle tracking [4] are two novel echocardiographic techniques that have been introduced for strain quantification. While both techniques have demonstrated promising potential for bedside regional function assessment at a high temporal resolution (> 250 frames/second), acquisition angle and operator dependence must be kept in mind while using these techniques.

Currently, cardiovascular magnetic resonance (CMR) tagging remains the reference standard for assessment of regional function [5]. It remains the most widely validated reproducible tool for multi-dimensional strain quantification.

### Techniques for Myocardial Tissue Tagging

Quantifying regional myocardial function, or regional deformation, originally required invasive surgical implantation of physical markers within the myocardium itself and then tracking their motion using external imaging. However, this method is impractical for clinical application and implanted markers tend to influence cardiac motion and thus distort the accuracy of measurements [6]. In 1988, Zerhouni et al. [7] introduced a magnetic resonance based noninvasive imaging method for tracking myocardial motion: myocardial tissue tagging. Noninvasive markers, known as tags, are created within the tissue by locally induced perturbations of the magnetization with selective radiofrequency saturation of multiple, thin tag planes in a plane perpendicular to the imaging plane prior to image acquisition. These perturbations then produce regions of reduced signal intensity that appear as dark lines in the acquired images [8]. Building upon this technique, Axel and Dougherty [9] then developed spatial modulation of magnetization (SPAMM) to allow the application of tags in two orthogonal directions that, combined, form a grid of sharp intrinsic tissue markers.

**Figure 1:**
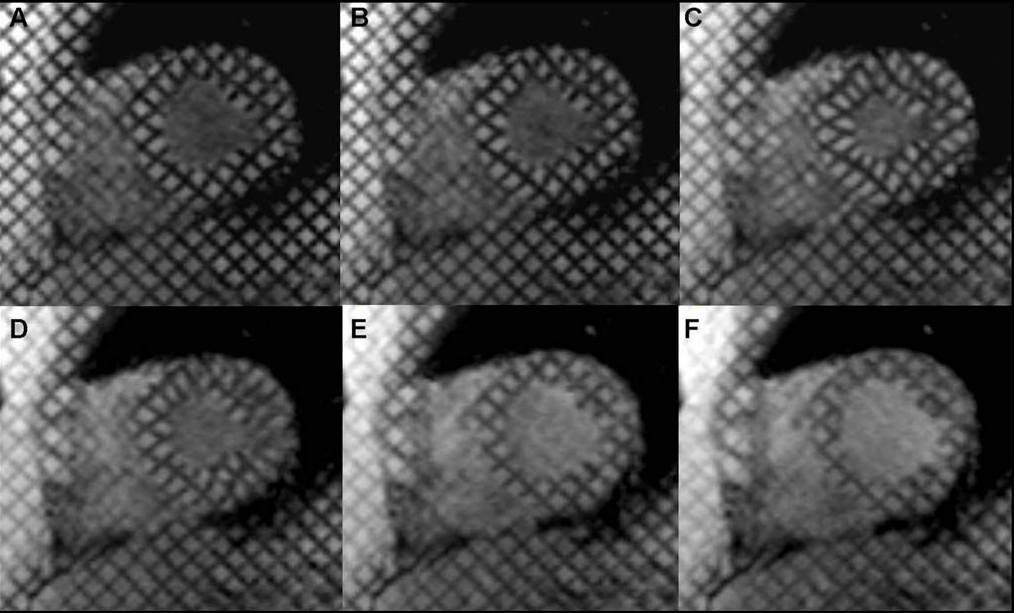
**Short axis tagging at the mid ventricular level covering the cardiac cycle (A-F)**. Tagging is applied upon detection of QRS complex at end diastole (A). Tag lines follow the myocardial deformation during systole (B, C, and D) and relax in diastole (E, F). Fading of tag lines occurs near end diastole (F) due to T1 tissue relaxation.

**Figure 2:**
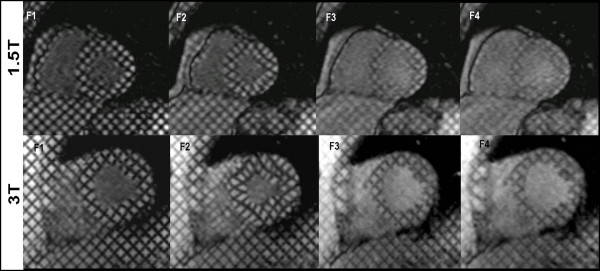
**1.5 and 3T mid ventricular short axis tagging covering the cardiac cycle (F1-F4)**. Note better tag-tissue contrast and longer tag persistence (F4) on 3T MRI system.

Initially designed to analyze myocardial contraction during systole, tags are typically created upon detection of the QRS complex of the electrocardiogram (ECG). The resulting tags then follow myocardial motion during the cardiac cycle, thus reflecting the underlying myocardial deformation (Figure 1). *See additional file *1: *Movie 1 demonstrating myocardial tagging covering the cardiac cycle*. However, fading of the tag lines close to end-diastole, as a result of T1 tissue relaxation, has limited its application to the systolic part of the cardiac cycle. Although spoiled gradient echo imaging is the commonly used sequence for tag generation at the widely available 1.5T magnets, recent studies [10,11] have proposed implementing steady state free precession (SSFP) to achieve better contrast and longer tag persistence. Using high field strength magnets for tagging acquisition may also reduce the problem of tag fading. In fact, despite the potential increase in susceptibility effects during cardiac imaging, applying myocardial tagging at higher field strength appears to provide a better contrast to noise ratio (CNR) as well as improve tag persistence. This could be attributed to a higher baseline signal to noise ratio (SNR) provided by 3T systems and prolongation of myocardial T1, thus improving the contrast between the tissue and the tag lines at end-diastole (Figure 2) [12]. Providing persistent tags allows quantification of cardiac strain evolution during late diastole, which can be used to assess diastolic myocardial dysfunction. Moreover, using the multi-planar capabilities of CMR, tag lines can be applied in the short or long axis cardiac planes to facilitate sophisticated three dimensional (3D) strain analysis.

In addition, current advances in gradient technology and acquisition techniques have greatly improved tagging temporal resolution. Currently, the most widely feasible temporal resolution is on the order of 15 - 20 msec, which is sufficient to detect peak systolic strain, the most extensively reported value in most clinical settings. However, a high temporal resolution actually becomes a critical issue especially when studying specific phases of the cardiac cycle that are characterized by rapid cardiac motion like the early diastolic filling phase in diastolic dysfunction, the early systolic emptying phase in conduction abnormalities as well as stress induced wall motion abnormalities [8]. Recently, parallel imaging techniques, such as simultaneous acquisition of spatial harmonics (SMASH) [13] and sensitivity encoding (SENSE) [14] have been proposed for speeding up the image acquisition. SENSE is the most widespread parallel imaging technique. Scan time reduction in SENSE benefits cardiac imaging leading to reduced breath-hold durations, or increased spatial resolution for a given breath-hold duration [8].

### Image Analysis

Although the tag line deformation in cine display can be followed and analyzed visually, this approach is subjective and limited by image quality. Therefore, quantitative analysis is preferred. Motion quantification techniques are divided into: a) differential optical flow-based methods that track motion by assessment of the temporal and spatial changes of image intensity; b) tag segmentation methods based on tracking of tag lines as in Findtags [15] and SPAMMVU [16] analysis; and, c) phase-based analysis methods which are the bases for Harmonic phase (HARP) analysis [17]. HARP analysis is currently the most widely used method for strain quantification since it is highly automated and, thus, limits both analysis time and subjective interference. Each tagging image is decomposed into a harmonic magnitude and a harmonic phase. The HARP method analyzes the motion in the tagging data by filtering the harmonic peaks in the frequency domain of the image. It computes a displacement map of the tag lines by tracking their phase changes through time. HARP can, thus, track the motion of a single point or of a whole myocardial segment (by taking the average of multiple adjacent points) through time to generate a dense regional dynamic color strain map throughout the cardiac cycle [18,19]*See additional file *2: *Movie 2 demonstrating color coded strain analysis of the tagged left ventricle using HARP; x-coordinate represents time frames, y-coordinate represents percent circumferential shortening (% E_cc_)*.

With the rising interest in computing 3D myocardial deformation maps, several techniques based on CMR tagging have been developed to quantify 3D strain through the acquisition of multiple orthogonal slices covering the whole volume of interest. The proposed methods used various 3D model-based approaches to reconstruct the 3D motion of the left ventricle (LV) from tagging images [6,20-22]. However, these methods predisposed to patient discomfort due to multiple breath holds required for data acquisition, in addition to image mis-registration caused by patient motion as well as heart rate variability from one slice to the other.

### Measurement of Deformation

Within the LV, the myofiber arrangement changes gradually from a right-handed helix in the subendocardium to a left-handed helix in the subepicardium, passing by a circumferential arrangement in the mid wall. Such complex fiber architecture results in complex patterns of deformation and changes in shape that are produced upon muscle contraction or relaxation [23]. Tagging analysis allows quantification of these multiplanar regional deformations and, in turn, offers a dynamic multidimensional measure of myocardial function.

#### Strain

Each element of strain is, simply, a measurement of the fractional or the percent change of length in a specific direction where *L*_*o *_is the original fiber length before tag deformation and *L *is the current length.

In 3D space, myocardial strain can be represented by two different coordinate systems: 1) The radial-fiber-crossfiber coordinate system, which is based on the fiber direction within the myocardial tissue and thus, requires a precise knowledge of fiber orientation angles [24], and 2) the radial-circumferential-longitudinal (RCL) coordinate system. In the later, circumferential strain (*E*_*CC*_) describes circumferential shortening in the short axis plane in a direction tangential to the epicardial surface; radial strain (*E*_*RR*_) describes myocardial thickening in a radial direction towards the center of the ventricle; and, longitudinal strain (*E*_*LL*_) represents base to apical shortening along the ventricular long axis. The RCL coordinate system is based on the cardiac geometry and, thus, is more convenient for clinical purposes [25].

**Figure 3:**
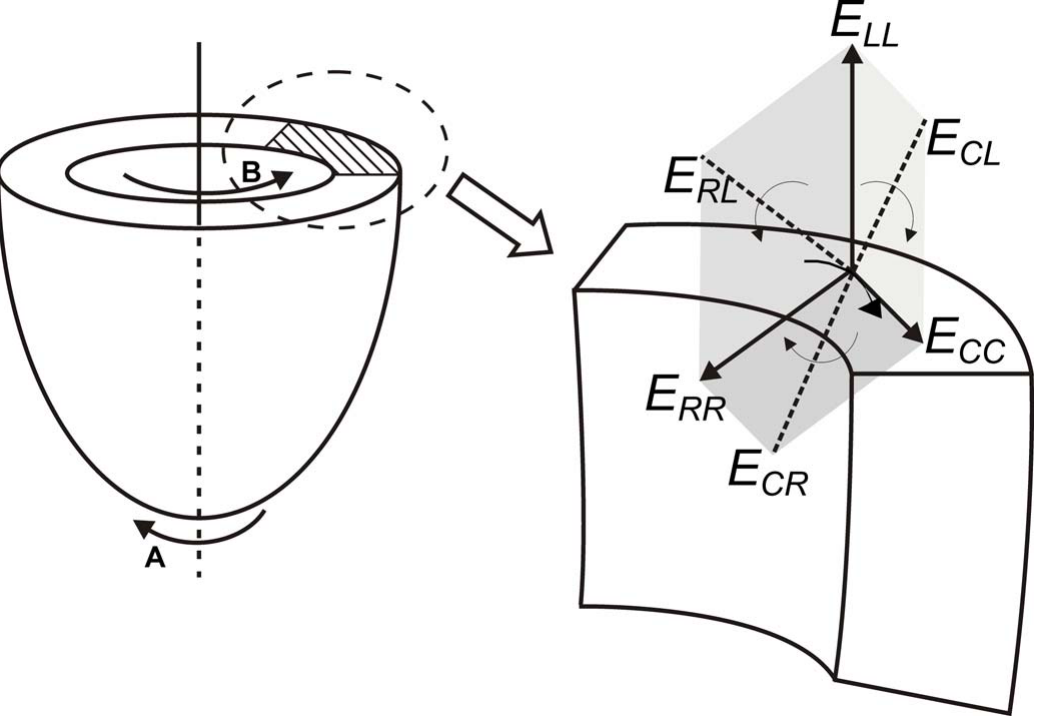
**Schematic diagram demonstrating the three dimensional circumferential - radial - longitudinal (RCL) coordinate system used for strain calculation**. Normal strains (dark solid arrows) are described with respect to the short axis plane: *E*_*CC *_represents circumferential shortening tangential to epicardial surface, *E*_*RR *_represents myocardial thickening radially towards the center of the ventricle and *E*_*LL *_represents basal to apical shortening along the ventricular long axis.*E*_*RC*_, *E*_*RL *_and *E*_*CL *_(dotted and curved arrows) represent change in angle caused by shear. Torsion represents the wringing motion caused by an apical counterclockwise rotation (curved arrow A) and a basal clockwise rotation (curved arrow B) around the ventricular long axis at end systole. Torsional deformation compensates for the opposing vectors in the subepicardium and subendocardium created by opposing myofiber arrangement in both layers.

In the RCL coordinate system, *E*_*CC*_, *E*_*RR*_, and *E*_*LL *_represent the normal strain elements of myocardial deformation in 3D space, whereas *E*_*RC*_, *E*_*RL*_, and *E*_*CL *_represent strain changes that occur in a plane between two of these three initially orthogonal normal directions known as shear strains [26]. The later component, *E*_*CL*_, represents the shear in the circumferential - longitudinal plane and is usually referred to as torsion (Figure 3).

Principal strains represent the maximum and minimum deformation occurring at a point in three orthogonal directions irrespective of cardiac geometry 1) *E*_1_, referred to as the maximum principal strain, represents the greatest elongation; 2) *E*_2_, the minimum principal strain, represents the greatest shortening; and, 3) *E*_3 _represents the strain occurring perpendicular to *E*_1 _and *E*_2 _Principal strains have been used in tagging studies [27] and have the advantage of representing the complex material deformation state regardless of cardiac geometry and the selected coordinate system. Similarly, the angles between the principal strain directions and the RCL coordinates have also been quantified: *α *represents the angle between *E*_2 _and *E*_*CC*_; *β *represents the angle between *E*_1 _and *E*_*RR *_[26].

#### Strain Rate

Strain rate (SR) represents the time derivative of strain values or, in other words, strain changes per unit time. Strain rate is calculated by taking the change in strain (S) between two time frames and dividing this by the time (t) difference between these two frames:

Using strain rate analysis for evaluation of LV diastolic function, Edvardsen et al. [28], revealed significant reduction of regional diastolic strain rate, reflecting regional diastolic dysfunction, among individuals with asymptomatic LV hypertrophy compared to healthy controls.

#### Torsion

During the systolic phase of each cardiac cycle, the contracting myofibers exert a wringing motion that assists in ejecting blood from the LV [24]. This wringing motion, as seen from the apex, is caused by basal clockwise rotation and apical counterclockwise rotation around the ventricular long axis (Figure 3). Torsion is directly related to the myofiber orientation and function such that earlier activation of the subendocardial helix in relation to the subepicardial helix, which is activated later in systole, may explain the brief initial rotation observed at the apex [29]. As well, twisting of the LV is associated with potential energy accumulation that is released during the isovolumetric relaxation phase by untwisting of the subendocardial fibers, thus contributing to diastolic suction [30,31]. Using CMR tagging for quantifying torsion in patients with aortic stenosis, Nagel et al. [32,33] found patients with severe stenosis to have reduced basal rotation and increased apical rotation during systole, along with delayed and prolonged untwisting during diastole. Thus, alteration in rotation patterns can also be used to discriminate subtle changes in systolic and diastolic dysfunction in the setting of a variety of cardiomyopathic states.

### Normal Left Ventricular Strain Patterns

Mid wall LV circumferential strain (*E*_*CC*_) is the most frequently computed parameter for quantifying regional function. This particular strain measure is favored, in part, due to myocardial geometry contributing an abundance of tagging data around the mid wall myocardial circumference compared to along the width of radial wall thickness [34]. This makes *E*_*CC *_data less sensitive to noise and more suitable for assessing the transmural strain gradient. In the normal heart, circumferential strain increases gradually from the base towards the apex. With respect to transverse regions, the greatest shortening is consistently observed in the anterior and lateral myocardial segments with the least deformation seen in the inferior wall. Additionally, *E*_*CC *_is seen to increase from epicardium towards endocardium. [34-36].

Maximum longitudinal LV strain (*E*_*LL*_) has also been measured in multiple studies [34-36] and is consistently greater at the apex compared to the base. This is contrary to longitudinal displacement, which is reportedly higher at the base, indicating greater basal translation caused by apical and mid ventricular pull during systole [24]. Similar to *E*_*CC*_, *E*_*LL *_shows a transmural pattern that increases from epicardium to endocardium. Maximum radial strain (*E*_*RR*_), on the other hand, shows a degree of disparity in its reported values with no consistent pattern across different studies [34]. Young et al. [36] reported higher *E*_*RR *_at the base particularly at the lateral segment, whereas Moore et al. [34] reported *E*_*RR *_increasing from base to apex. However, endocardial *E*_*RR *_was consistently higher than epicardial *E*_*RR *_Congruent with the general pattern of almost all other measures, maximum torsion angle also increases across the LV wall from epicardium towards the endocardium as well as from the base toward the apex in the normal heart.

With respect to the regional timing of myocardial contraction, Zwanenburg et al. [37] used high temporal resolution (14 ms) CMR tagging to examine spatial patterns of circumferential systolic shortening during the cardiac cycle. They observed the earliest onset of circumferential systolic shortening in the lateral wall and the latest onset in the septum. Conversely, peak systolic shortening was reached earlier in the septum compared to the lateral wall. When applying the same technique to assess both diseased and healthy myocardium, no consistent pattern of contraction propagation was detected in ischemic compared to normal subjects [38].

When referring to expected normal patterns of regional myocardial deformation, consideration should always be made for the effects of aging. Fonseca et al. [39] and Oxenham et al. [40] observed that aging was associated with little change in peak circumferential or longitudinal shortening. However, they noted relatively prolonged time to peak shortening in older compared to younger subjects. Interestingly, older age groups, compared to younger subjects, also demonstrated higher peak rotation that persisted for longer. As well, aging related diastolic dysfunction was evident in the form of reduced peak rate of relaxation of *E*_*CC *_and *E*_*LL *_(*p *< 0.001 for both). Using 2D displacement data from tagging to examine differences in the transmural distribution of myocardial shortening, Lumens et al. [41] further observed loss of contractile myofiber function in the subendocardium relative to the subepicardium among asymptomatic, aged subjects.

### Coronary Artery Disease

Many individuals with underlying coronary artery disease are asymptomatic and go unnoticed before they present with major coronary events. Since atherosclerosis is the most important primary etiologic factor predisposing to the development of HF, the Multi-ethnic Study of Atherosclerosis (MESA) was initiated in 2000 to investigate the prevalence, correlates, and progression of subclinical cardiovascular disease in a community-based population of 6,500 men and women of different racial/ethnic backgrounds. Harmonic phase analysis of myocardial tagged images proved highly reproducible in MESA for detecting silent myocardial dysfunction in asymptomatic populations [42]. Using CMR tagging, investigators studied the relationship between regional LV function and traditional risk factors for atherosclerosis, such as hypertension and smoking. Increased diastolic blood pressure and smoking were associated with lower *E*_*CC *_in the left anterior descending and right coronary territories, with dose dependent effect seen between LV function and smoking [43]. With respect to the structural changes associated with long-standing hypertension, in particular, asymptomatic MESA individuals with LV hypertrophy had reduced early diastolic regional function, quantified using diastolic strain rate (*p *< 0.001). This finding remained significant even in the setting of preserved regional systolic function [28], supporting the concept of hypertensive heart disease and diastolic dysfunction as potential precursors to clinical HF.

Beyond their association with cardiovascular risk factors, changes in regional myocardial function also correlated with more specific markers of subclinical atherosclerosis in MESA. Coronary artery calcification burden, quantified by computed tomography, was related to reduced regional *E*_*CC *_and its strain rate in the corresponding coronary territory, suggesting a link between coronary atherosclerosis and incipient regional dysfunction [44]. Increased carotid wall stiffness and intima-media thickness (IMT) were also strongly related with reduced systolic and diastolic regional function (*E*_*CC*_) among individuals free of known cardiovascular disease [45,46]. In particular, Fernandes et al. [46] showed that increased IMT was related to less circumferential shortening in all myocardial regions (*p *< 0.05) except in the inferior wall. In addition, greater IMT was associated with a lower diastolic strain rate, representing reduced diastolic function, in all regions (*p *< 0.01) except the anterior wall.

### Myocardial Ischemia

Given the strong association between regional myocardial dysfunction and markers of prevalent coronary disease, multiple studies have used tagging to further explore its relation with myocardial ischemia. Rosen et al. [47] examined the relationship between regional coronary perfusion reserve and regional myocardial function in a subset of 74 symptom-free MESA participants who underwent adenosine stress CMR perfusion scans. In this study, reduced coronary perfusion reserve was associated with reduced regional systolic function mainly in the right coronary and left circumflex territories.

A particularly useful feature offered by myocardial tagging is its ability to provide a detailed assessment of contractile reserve in the setting of ischemia. Myocardial tagging, either independently or combined with the findings of gadolinium enhanced CMR, can also assess and predict the extent of functional recovery after successful reperfusion of a transmural myocardial infarction [48,49]. As well, myocardial CMR tagging is particularly advantageous over plain cine imaging for quantifying contractile reserve in the setting of dobutamine stress CMR. In fact, combining tagging with dobutamine stress for detecting chronic hibernating myocardium yielded a sensitivity of 89% and a specificity of 93% for recovery of segmental function 4-8 weeks after revascularization in 10 patients [50]. Furthermore, assessing contractile reserve across the myocardial layers is possible with tagging given its superior spatial resolution and higher tissue characterization [51].

When comparing qualitative assessment of myocardial contractile reserve by CMR tissue tagging versus echocardiography, CMR had a similar sensitivity (82% versus 86%) but lower specificity (69% versus 87%). Overall accuracy was 76% for CMR and 85% for echo [52]. Lower CMR specificity could be attributed to difficulties in cross registration between the two modalities [53]. In 211 patients with chest pain, Kuijpers et al. [54] showed that dobutamine CMR with myocardial tagging could detect more angiographically confirmed new wall motion abnormalities (68 patients) than dobutamine CMR without tagging (58 patients). Moreover, myocardial tagging demonstrated better ability to discriminate between patients at risk for major adverse cardiac events and those who were more likely to remain event-free.

### Myocardial Infarction

**Figure 4:**
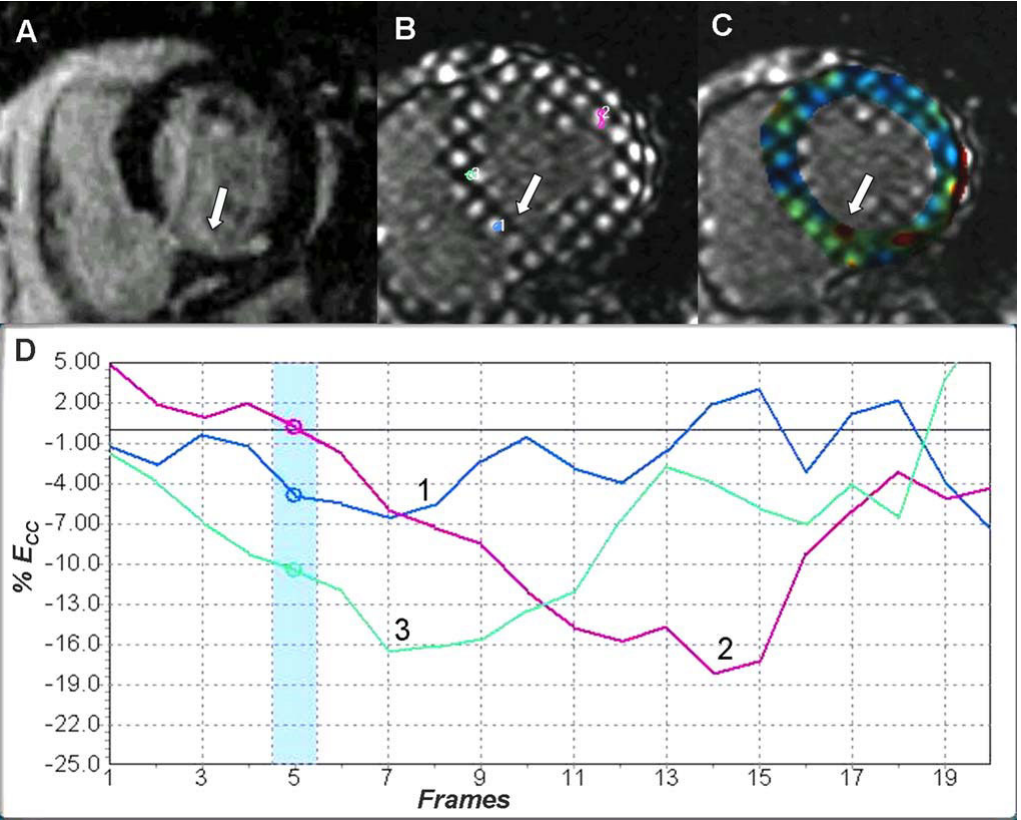
**Myocardial infarction at the infero-septal region of left ventricle (A-D)**. Inversion recovery mid ventricular short axis image (A) demonstrates subendocardial delayed enhancement at the infero-septal region of the left ventricle (white arrow) in the distribution of right coronary artery. Corresponding tagged image and strain analysis curves (B & D) demonstrate reduced *E*_*CC *_at the infarct region (blue curve 1) compared to lateral wall (red curve 2) and adjacent non enhanced myocardium (green curve 3). Color coding (C) of tagged image aids visual assessment of regional dysfunction (dysfunctional infarcted myocardium in green). See additional file 3: Movie 3 for the original data used.

In addition to assessing contractile reserve in the setting of ischemia, myocardial tagging has made it possible for investigators to elucidate the mechanisms related to post-infarct ventricular remodeling. In particular, myocardial tagging is able to define changes in regional function involving the infarcted myocardium versus areas remote from the infarcted myocardium (Figure 4 and additional file 3). In the acute post-infarct period, alteration and reorientation of principal strains have been observed in both the infarct and remote myocardium in animal models and humans [55,56]. Kramer et al. [57] found reduced circumferential and longitudinal segmental shortening in adjacent compared to remote myocardium, reflecting the probable effect of increased wall stress on LV remodeling. Nagel et al. [33] studied 18 patients with anterolateral wall infarction and found reduced apical systolic rotation as well as delayed and prolonged diastolic untwisting in these patients compared to healthy controls. Moreover, infarcted myocardium in the setting of microvascular obstruction demonstrated reduced regional systolic circumferential and longitudinal shortening in relation to the extent of obstruction [58]. Overall, when comparing strain analysis to wall thickening assessment as two different techniques for assessing post-infarcted myocardium, 2D strain quantification was more accurate in discriminating infarct from remote myocardium [59].

### Non-Ischemic Cardiomyopathies

***Non-ischemic dilated cardiomyopathies (DCM) ***are characterized by ventricular eccentric remodeling, increased ventricular volume, and globally reduced ventricular function. In a cohort of 13 DCM patients, Young et al. [60] quantified 3D motion and myocardial strain with the aid of a finite element model. Five of the patients were followed up after LV reduction by partial left ventriculotomy (PLV). In the examined subjects, circumferential and longitudinal systolic lengthening (i.e. reduced systolic function) occurred in the septum whereas the lateral wall demonstrated normal systolic shortening. Increased wall stress may have contributed to this observed heterogeneity of regional function. Post PLV, patients demonstrated significant recovery of their septal function, whereas lateral wall shortening was significantly reduced (*p *< 0.02 pre vs. post). Kanzaki et al. [61] studied another cohort of 26 DCM subjects and observed that peak systolic torsion was reduced in DCM cases compared to controls at both the base (0.1 ± 2.9° vs. 2.6 ± 1.6°, *p *< 0.05) and apex (-5.9 ± 5.3° vs. -11.2 ± 2.5°, *p *< 0.001), whereas the timing of peak rotation was earlier (66 ± 22 vs. 104 ± 16% systole, *p *< 0.001). Systolic torsion was characterized by a discontinuing counter rotation of the apex with respect to the base before end-systole. In addition, amplitude of peak systolic torsion was impaired in proportion to the degree of LV dysfunction. In a trial to assess cardiac mechanics and clinical outcome of PLV, 24 DCM patients underwent CMR tagging studies before and then 3 and 12 months after PLV. Circumferential LV shortening at three short axis levels was increased at both post-surgical time points. Thus, using these and similar methods, CMR tagging can serve as a useful tool for assessing the efficacy of various surgical as well as non-surgical interventions for DCM [62].

***Hypertrophic cardiomyopathy (HCM) ***is the most frequently occurring genetic cardiomyopathy responsible for sudden cardiac death in young trained athletes. Underlying histological changes include myofibrillar disarray and abnormal intramural coronary vasculature [63]. While studying 3D myocardial mechanics in HCM, Young et al. [36] and Dong et al. [64] observed reduced 3D regional myocardial shortening but also greater LV torsion in cases compared to controls (19.9 ± 2.4° vs. 14.6 ± 2.7°, *p *< 0.01). Changes in myocardial contractility in the non-hypertrophied regions of HCM have been a subject of controversy. Therefore, to assess regional function in these non-hypertrophied regions, Mishiro et al. [65] examined 20 patients with asymmetric septal hypertrophy and showed that regional contractility was impaired in both hypertrophied and non-hypertrophied regions. They also observed systolic LV wall asynchrony. Implementing a modified CSPAMM sequence that allows full cardiac cycle data acquisition using the cardiac phase to order reconstruction technique (CAPTOR), Ennis et al. [66] compared mid wall *E*_*CC *_during systole and diastole in 8 HCM patients and 6 normal volunteers. In HCM patients, total systolic strains were reduced in the septal and inferior regions (*p *< 0.01 for both). On the other hand, early diastolic strain rates were significantly reduced in all regions, indicating slow and impaired filling function in HCM cases relative to controls.

Myocardial tagging has also been used to explore the relation of regional dysfunction with areas of abnormal myocardial perfusion in HCM. In a prospective trial including 53 patients, Soler et al. [67] found good correlation between delayed enhancement areas in hypertrophic cardiomyopathy and perfusion defects (r = 0.5, *p *< 0.01), whereas a weak but significant correlation was detected between delayed enhancement and hypokinetic segments. Recently, Kim et al. [68] demonstrated impaired circumferential shortening in delayed enhancement areas, where *E*_*CC *_was more substantially reduced in the regions displaying focal nodular enhancement patterns rather than in patchy enhanced regions.

***Muscular dystrophies ***are a group of inherited diseases involving primarily the skeletal muscle leading to degeneration and progressive weakness. Cardiac involvement is not infrequent and can manifest as cardiomyopathy, conduction defects, and sudden death. In a study conducted by Ashford et al. [69], regional myocardial function was assessed using CMR myocardial tagging in 13 patients with Duchenne muscular dystrophy without clinically apparent heart diseases. Despite preserved LV volumes, ejection fraction, and torsion, these patients manifested reduced basal and mid-ventricular circumferential strain compared to healthy subjects. Thus, CMR with tissue tagging can be used as a sensitive tool for detecting occult myocardial dysfunction related to muscular dystrophies that might otherwise go unnoticed.

### Ventricular Dyssynchrony

Cardiac resynchronization therapy (CRT) is the treatment of choice for management of patients with ventricular dyssynchrony [70]. Despite its overall efficacy, 20-30% of the patients receiving CRT do not show favorable improvement. This has highlighted the need to improve prospective identification of the optimal candidates for such treatment. Current approaches to identifying candidate patients for CRT rely on assessing QRS duration, where QRS prolongation is often an indicator of underlying dyssynchronous ventricular contraction. However, the duration of the QRS interval has been proven inadequate for predicting post-CRT acute and chronic responses to therapy. Therefore, several echocardiographic methods have been proposed to aid in assessing and selecting patients for CRT. Echocardiographic techniques, however, are greatly limited by the operator performance as well as the availability of acoustic windows for transducer placement [71]. On the other hand, quantitative CMR strain analysis offers a highly reproducible, operator independent, 3D regional myocardial activation analysis that offers a better suited technique for evaluating dyssynchrony. Using CMR strain analysis, the difference in mechanical activation (T_onset_) and time to peak contraction (T_peak_) between the septum and lateral wall can be easily derived from all segments in a given cross section [37,38]. Similarly, the variance of strain magnitude can be easily determined for each short axis slice and can be averaged from base to apex [72]. Moreover, several additional metrics have been derived [72-74] and employed to determine the optimal LV pacing region for CRT [75] and predict the response to therapy. Combining CMR tagging data with delayed enhancement scar quantification further improves predictive accuracy [76]. Future studies promise to clarify which of these CMR tagging based metrics may offer the most predictive information with respect to benefit from CRT.

### Right Ventricular Tagging

Right ventricular (RV) function plays a pivotal prognostic role in a wide variety of diseases involving the heart as well as the lung. Thus, the application of CMR tagging has been extended to include assessments of RV regional function. It should be noted, however, that there are several technical challenges involved in applying CMR tagging analyses to the RV. For example, the very thin wall (<5 mm) of the normal RV offers less than the minimum optimal tag spacing (≥6 mm) and therefore limits the number of tag stripes that would be required for accurate quantification [77]. Nonetheless, while making attempts to accommodate for this limitation, a number of CMR myocardial tagging studies have been performed on the healthy and diseased RV to investigate patterns of mechanical deformation.

**Figure 5:**
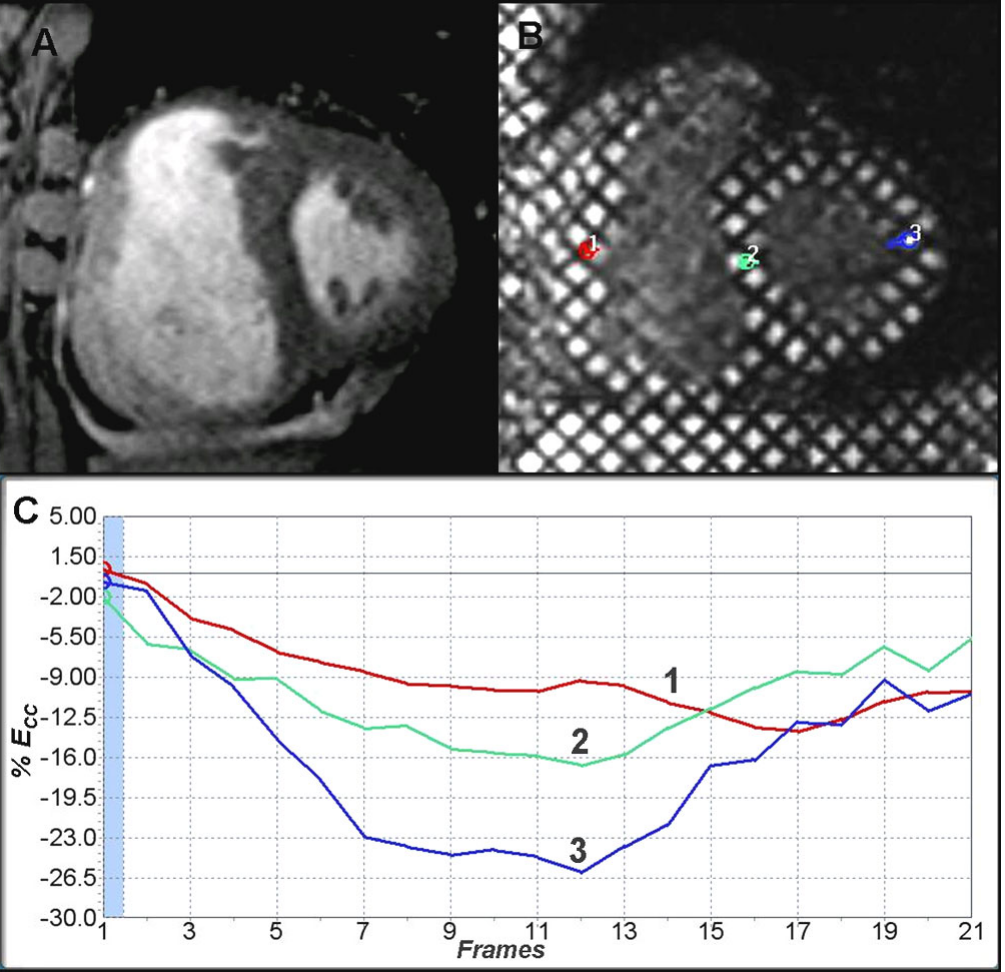
**Pulmonary Arterial Hypertension (A-C)**. Cine gradient echo short axis image (A) showing right ventricular hypertrophy and systolic flattening of the inter-ventricular septum in a 64 year old patient with pulmonary arterial hypertension. Corresponding tagged image (B) and graph (C) show reduced magnitude and delayed RV free wall peak shortening (red curve 1) compared to the septum (green curve 2) and LV lateral wall (blue curve 3).

In light of the challenges associated with applying CMR tagging to the RV, several novel approaches have been developed. Using measures of percent segmental shortening, Klien et al. [78] observed that regional RV free wall systolic function increased monotonically from base (12%) to mid (14%) to apical slices (16%). Young et al. [79] were first to use a finite element model for 3D reconstruction of the in-plane deformations of the RV free wall. Using this model, they were able to quantify all the components of the in-plane strain tensor in the RV free wall. Fayad et al. [77] then designed a specific breath hold imaging sequence with 1D tag lines for RV tagging acquisition and, thus, were able to characterize the RV regional deformation in 7 patients with pulmonary hypertension and 10 healthy controls. In the normal subjects, regional RV circumferential shortening was non-uniform, with increased shortening observed mainly in the RV free wall progressing from the outflow tract towards the apex. A more complex non-uniform pattern was also noted in the longitudinal direction, where greatest shortening was also seen at the RV out flow tract. In pulmonary hypertension patients, both circumferential and longitudinal shortening were globally reduced with significant reduction observed at the outflow tract. These findings were conjectured as possibly related to increased stress at the RV outflow tract region as it plays a resistive role in preventing transmission of high pressures from the RV free wall to the pulmonary circulation [80]. Interestingly, Vonk Nordegraaf et al[81] recently used CMR myocardial tagging to demonstrate interventricular mechanical asynchrony between the RV and LV in pulmonary hypertension patients caused by prolongation of RV systolic contraction due to increased pressure overload (Figure 5). Therefore, further developments in analyzing regional function of the RV will likely facilitate more detailed studies of interventricular performance.

## Conclusion

In conclusion, CMR tissue tagging is a powerful non-invasive diagnostic tool for quantifying regional systolic and diastolic myocardial function. Owing to the inherent features of CMR tissue characterization and its multiplanar capabilities, CMR tagging is able to reveal previously undetected components of regional myocardial mechanical function and, thus, aid in the early detection and management of a wide range of myocardial disease processes. Moreover, ongoing developments in the technology, imaging techniques, and analytical tools used to implement CMR tagging will likely further advance current capabilities for performing sophisticated analyses of regional myocardial function. As such, myocardial tagging promises to continue to enhance our understanding of the mechanical complexities underlying the function of the normal and pathological heart

## Competing interests

Dr Nael F. Osman is a founder and shareholder in Diagnosoft Inc. The terms of this arrangement have been approved by the Johns Hopkins University in accordance with its conflict of interest policies.

## Authors' contributions

MS and SC drafted the initial review article, MS performed the literature search, NO, DB and JL participated in the article organization, editing and final drafting of the manuscript. All authors read and approved the final manuscript.

## List of abbreviations

CMR: cardiovascular magnetic resonance; HF: heart failure; SPAMM: spatial modulation of magnetization; CSPAMM: complementary spatial modulation of magnetization; ECG: electrocardiogram; SSFP: steady state free precession; CNR: contrast to noise ratio; SNR: signal to noise ratio; 2D: Two dimensional; 3D: three dimensional; HARP: harmonic Phase; LV: left ventricle; RV: right ventricle; SR: strain rate; MESA: multi-ethnic study of atherosclerosis; IMT: intima-media thickness; PLV: partial left ventriculotomy; DCM: dilated cardiomyopathy; HCM: hypertrophic cardiomyopathy; CRT: cardiac resynchronization therapy.

## Supplementary Material

Additional file 1 **Movie 1**. Demonstrating myocardial tagging covering the cardiac cycle Click here for file

Additional file 2 **Movie 2**. Demonstrating color coded strain analysis of the tagged left ventricle using HARP; x-coordinate represents time frames, y-coordinate represents percent circumferential shortening (% Ecc) Click here for file

Additional file 3 **Movie 3**. Original data used Click here for file
